# Neuroprotective Effects of Dexmedetomidine on the Ketamine-Induced Disruption of the Proliferation and Differentiation of Developing Neural Stem Cells in the Subventricular Zone

**DOI:** 10.3389/fped.2021.649284

**Published:** 2021-07-27

**Authors:** Huanhuan Sha, Peipei Peng, Guohua Wei, Juan Wang, Yuqing Wu, He Huang

**Affiliations:** ^1^Department of Anesthesiology and Perioperative Medicine, First Affiliated Hospital of Nanjing Medical University, Nanjing, China; ^2^Jiangsu Province Key Laboratory of Anesthesiology, Xuzhou Medical University, Xuzhou, China

**Keywords:** dexmedetomidine, neural stem cells, subventricular zone, neuroprotection, ketamine, olfactory cognitive function

## Abstract

**Background:** Ketamine disrupts the proliferation and differentiation of developing neural stem cells (NSCs). Therefore, the safe use of ketamine in pediatric anesthesia has been an issue of increasing concern among anesthesiologists and children's parents. Dexmedetomidine (DEX) is widely used in sedation as an antianxiety agent and for analgesia. DEX has recently been shown to provide neuroprotection against anesthetic-induced neurotoxicity in the developing brain. The aim of this *in vivo* study was to investigate whether DEX exerted neuroprotective effects on the proliferation and differentiation of NSCs in the subventricular zone (SVZ) following neonatal ketamine exposure.

**Methods:** Postnatal day 7 (PND-7) male Sprague-Dawley rats were equally divided into the following five groups: control group (*n* = 8), ketamine group (*n* = 8), 1 μg/kg DEX+ketamine group (*n* = 8), 5 μg/kg DEX+ketamine group (*n* = 8) and 10 μg/kg DEX+ketamine group (*n* = 8). Immediately after treatment, rats received a single intraperitoneal injection of BrdU, and the proliferation and differentiation of NSCs in the SVZ were assessed using immunostaining at 24 h after the BrdU injection. In the olfactory behavioral tests, rats in each group were raised until 2 months old, and the buried food test and olfactory memory test were performed.

**Results:** The proliferation of NSCs and astrocytic differentiation in the SVZ were significantly inhibited at 24 h after repeated ketamine exposure in the neonatal period, and neuronal differentiation was markedly increased. Furthermore, pretreatment with moderately high (5 μg/kg) or high doses (10 μg/kg) of DEX reversed ketamine-induced disturbances in the proliferation and differentiation of NSCs. In the behavior tests, repeated neonatal ketamine exposure induced olfactory cognitive dysfunction in the adult stage, and moderately high and high doses of DEX reversed the olfactory cognitive dysfunction induced by ketamine.

**Conclusions:** Based on the present findings, pretreatment with a moderately high (5 μg/kg) or high dose (10 μg/kg) of DEX may alleviate the developmental neurogenesis disorder in the SVZ at 24 h after repeated ketamine exposure and improve olfactory cognitive dysfunction in adulthood.

## Background

Ketamine is a widely used analgesic and sedative during pediatric examinations and surgical operations (1, 2). Recently, a subanesthetic dose of ketamine has been confirmed to produce rapid acting and sustained antidepressant effects on patients with major depressive disorder (3). However, an increasing number of studies have suggested that neonatal ketamine exposure might cause neuroapoptosis, disturb normal neurogenesis in the developing brain and increase the risk of delayed neurocognitive dysfunction (4–7). Evidence from clinical studies also supports the hypothesis that ketamine exerts long-term adverse effects on neurocognitive function in children and infants (1, 8). Relevant research conclusions have prompted anesthesiologists to re-evaluate the safety of using ketamine anesthesia in pediatric patients and to search for possible protective measures.

Dexmedetomidine (DEX), a highly selective α_2_-adrenoceptor agonist, is a widely used anxiolytic, sedative and analgesic in clinical pediatric anesthesia and intensive care (9, 10). DEX exerts protective effects on vital organs, including decreases in lung and kidney damage and neural apoptosis (11, 12). In anesthesia models using neonatal animals, DEX has been suggested to protect against inhalation anesthetic-induced neurotoxicity in the developing brain (13, 14). In clinical pediatric anesthesia, a medication strategy with DEX is increasingly accepted as a method to reduce the adverse effects of ketamine (15, 16). However, the potential neuroprotective pathway of DEX requires further investigation.

Neurogenesis in the hippocampus and subventricular zone (SVZ) is an important process in the developing brain (17, 18). Early disruption of these regions has the potential to exert an adverse effect on the formation of neural circuits (19). Currently, little is known about whether DEX pretreatment exerts protective effects on ketamine-induced neurotoxicity in the SVZ. Therefore, the purpose of this study was to investigate the protective effects of DEX on neurogenesis in the SVZ in a repeated ketamine exposure model using neonatal rats.

## Materials and Methods

### Animals and Drug Administration

All animal procedures were approved by the Institutional Animal Care and Use Committee of Nanjing Medical University. Timed-pregnant Sprague-Dawley rats were housed at 24°C on a 12-h light/dark cycle with free access to food and water. Postnatal day 7 (PND-7) male rats (11–14 g) were selected from all the pups and used in the experiments.

Ketamine and dexmedetomidine (DEX, HengRui Pharma, China) were dissolved in normal saline (0.9% NaCl). The rat were randomly divided into five groups: (1) control group (CON): the rats received an equal volume of saline (*n* = 8); (2) ketamine group (KET): the rats were administered four intraperitoneal injections of 40 mg/kg ketamine at 1-h intervals (40 mg/kg × 4) (*n* = 8); (3) KET+DEX 1 group: the rats were pretreated with an intraperitoneal injection of 1 μg/kg DEX 30 min prior to ketamine anesthesia (40 mg/kg × 4) (*n* = 8); (4) KET+DEX 5 group: the rats were pretreated with an intraperitoneal injection of 5 μg/kg DEX 30 min prior to ketamine anesthesia (40 mg/kg × 4) (*n* = 8); and (5) KET+DEX 10 group: the rats were pretreated with an intraperitoneal injection of 10 μg/kg DEX 30 min prior to intraperitoneal ketamine anesthesia (40 mg/kg × 4) (*n* = 8). Hypothermia and hypoxia often occur in anesthetized rat pups, and hypothermia and hypoxia stress may be closely associated with adverse reactions and even lead to the death of rat pups. In the present study, custom-made temperature probes were used to control the temperature at 36.5 ± 1°C using computer-controlled heater/cooler plates integrated into the chamber floor. The rat pups were returned to their chamber to help maintain their body temperature between each ketamine injection. Meanwhile, the oxygen concentration in the chamber was maintained at 50%. During the experiment, we carefully observed the skin color and breathing status of each rat pup. Four injections of 40 mg/kg ketamine administered at 1 h intervals produced a satisfactory level of anesthesia, and all animals in each group survived after anesthesia.

In order to ensure that hypoxia did not occur in the newborn animal during anesthesia, arterial blood gas levels were measured. We performed the open-chest operation during the ketamine anesthesia and the heart was isolated, then we punctured the left ventricle with a 30-gauge needle under direct vision and collected 0.25 mL arterial blood (six animals per group). Arterial blood gases were measured with a portable clinical analyzer (GEM Premier 3000, USA).

### BrdU Injections

Immediately after the treatment of each group, the rats received a single intraperitoneal injection of BrdU (5-bromo-2-deoxyuridine; Sigma, 100 mg/kg) in a 0.9% NaCl solution. 34 h after the BrdU injection, the animals were intraperitoneally injected with 4% chloral hydrate (0.1 mL/10 g) and the rat pups transcardially perfused with 4% paraformaldehyde/0.1 M PBS. The detailed experimental protocol is described in Table 1.

**Table 1:** Experimental protocol.

**Targeted Process**	**BrdU Injections Timing/Dose (mg/kg)**	**Interval to Perfusion**	**IF Stain**
NSC proliferation	PND-7/100	24 h (PND-8)	BrdU
Neuronal differentiation	PND-7/100	24 h (PND-8)	β-tubulin III/BrdU
Astrocytic differentiation	PND-7/100	24 h (PND-8)	GFAP/BrdU

*The interval to perfusion refers to the time from the BrdU injection to transcardiac perfusion. IF, immunofluorescence*.

### Tissue Preparation and Immunofluorescence Staining

Double immunofluorescence staining with BrdU was conducted as described in our previously reported methods (6). The rat brain was isolated, removed and fixed with 4% paraformaldehyde fixative for 6 h. Then, the brain was embedded in OCT medium on ice and stored at −80°C. Coronal brain sections were cut using a microtome at a thickness of 30 μm. The SVZ in the coronal brain sections including the 0.40 mm rostral and −0.20 mm caudal to bregma were selected for BrdU single-label immunofluorescence staining and β-tubulin III/BrdU and GFAP/BrdU double-label immunofluorescence staining, respectively. (www.ial-developmental-neurobiology.com). The selected sections were incubated with 50% formamide in PBS for 2 h at 65°C prior to incubation with 2 N hydrochloric acid for 30 min at 45°C and three washes with PBS for 10 min. Non-specific epitopes were blocked with 10% donkey serum in PBS containing 0.3% Triton-X for 2 h prior to overnight incubation at 4°C with the appropriate primary antibody (Table 2) in PBS containing 0.3% Triton-X. After three washes with PBS, the sections were incubated with suitable fluorescent dye-conjugated secondary antibodies (Alexa Fluor 488-labeled donkey anti-rabbit and Alexa Fluor 594-labeled donkey anti-mouse; 1:200; Invitrogen) for 2 h at room temperature. The sections were observed, and image stacks were captured by a skilled pathologist who was blinded to the groups using a laser scanning confocal microscope (Fluoview 1000, Olympus). For each brain, we selected three sections for immunofluorescence staining for BrdU, three sections for immunofluorescence staining for β-tubulin III/BrdU and three sections for immunofluorescence staining for GFAP/BrdU.

**Table 2:** Primary antibodies.

**Raised Against**	**Supplier**	**Raised in**	**Dilution**
BrdU	Sigma	Mouse, monoclonal	1:1000
GFAP	Millipore	Rabbit, monoclonal	1:200
β-tubulin III	Abcam	Rabbit, polyclonal	1:100

*List of primary antibodies, their suppliers, the animal used to raise the antibodies in, and the working dilution*.

The positive cells in the SVZ were manually counted by a skilled pathologist who was blinded to this research with the help of the cell counter function of ImageJ 1.4 software (National Institutes of Health, Bethesda, MD, USA). One SVZ slice randomly selected from each animal was used to calculate the number of BrdU^+^ cells, the ratio of β-tubulin III^+^/BrdU^+^ cells to BrdU^+^ cells in a randomly selected SVZ slice and the ratio of GFAP^+^/BrdU^+^ cells to BrdU^+^ cells in a randomly selected SVZ slice were calculated respectively (*n* = eight animals/group).

### Behavioral Testing

The male PND-7 rats in each group were raised until 2 months old and the olfactory behavioral tests were conducted, including the buried food test and olfactory memory test.

### Buried Food Test

The buried food experiment is one of the most classic olfactory behavioral tests. This task is used to test the ability of animals to detect odors. The experimental device was a 35 × 20 × 15 cm cage in which 5 cm padding was laid. Then, feed was randomly buried 1.5 cm below the surface of the padding. All testing occurred between 10:00 a.m. and 1:00 p.m. During three consecutive training days, the 2-month old rats were placed in the experimental apparatus and allowed to habituate themselves to the environment for 5 min. The testing phase then followed an intertrial interval. In the formal experiment, all rats were fasted for 12 h. The rats were returned to the apparatus and allowed to freely explore the cage to detect feed. We recorded the time taken for rats to dig out and eat the feed. If the rat could not find the feed within 5 min, the rat failed in the search test, and the search time was recorded as 5 min. The feed was buried at a random location each day, and the average value of 3 consecutive days was recorded as the final experimental result (*n* = 10 animals/group).

### Olfactory Memory Test

Olfactory memory tests comprehensively investigate the ability of rats to recognize and remember smells. The experimental device was a 55 × 30 × 25 cm cage divided into three equally sized compartments by two opaque partitions with a 5 × 5 cm gap at the bottom of each partition. Filter paper dipped in 10 μL of cinnamon essential oil or 10 μL of ginger essential oil was placed in each compartment on the left and right sides. All rats were fasted for 12 h before the experiment. On the 3 training days, the feed was placed in the compartment where the ginger essential oil was located, and then the rats were placed in the middle compartment and allowed to freely explore the feed for 5 min. During the interval between each exploration, alcohol was used to clean the experimental device. In the testing phase, no feed was placed in the compartments where the ginger essential oil was located, the rat was placed in the middle compartment, and the time spent exploring each compartment within 5 min was recorded (*n* = 10 animals/group).

### Statistical Analysis

The data obtained from the analysis of the proliferation and differentiation of NSCs are reported as medians and interquartile ranges (IQRs). The blood gas levels are presented as means ± SD. Differences among the treatment groups were evaluated using one-way ANOVA. The statistical analysis was performed and the graphs were generated using GraphPad Prism 5 software. Significant differences between the groups were analyzed using one-way ANOVA. *P* < 0.05 was considered statistically significant.

## Results

### Arterial Blood Gas Analyses

In the experiment, all rats in each group survived to the end of the experiment. The animals' skin was ruddy, and the respiration was smooth. No significant changes in pH, pCO_2_ or pO_2_ were observed between the groups (Table 3). It was suggested that pup rats were not in the hypothermia and hypoxia during anesthesia.

**Table 3:** The arterial blood gases analyse.

**Parameter**	**Control group**	**Ketamine group**	**KET+DEX 1 group**	**KET+DEX 5 group**	**KET+DEX 10 group**
pH	7.39 ± 0.02	7.41 ± 0.03	7.41 ± 0.03	7.42 ± 0.04	7.42 ± 0.04
${\text{P}}_{\text{CO}2}^{\text{a}}$ (mmHg)	39.3 ± 1.4	39.1 ± 1.7	38.0 ± 2.1	40.0 ± 2.3	39.8 ± 2.6
${\text{P}}_{\text{CO}2}^{\text{b}}$ (mmHg)	168.0 ± 3.1	166.0 ± 4.4	163.3 ± 4.5	164.8 ± 5.1	162.2 ± 4.0

a *Pco_2_ pressure carbon dioxide*.

b *Po_2_ pressure oxygen*.

### Effects of Ketamine on the Proliferation and Differentiation of NSCs in the SVZ

**Figure 1:**
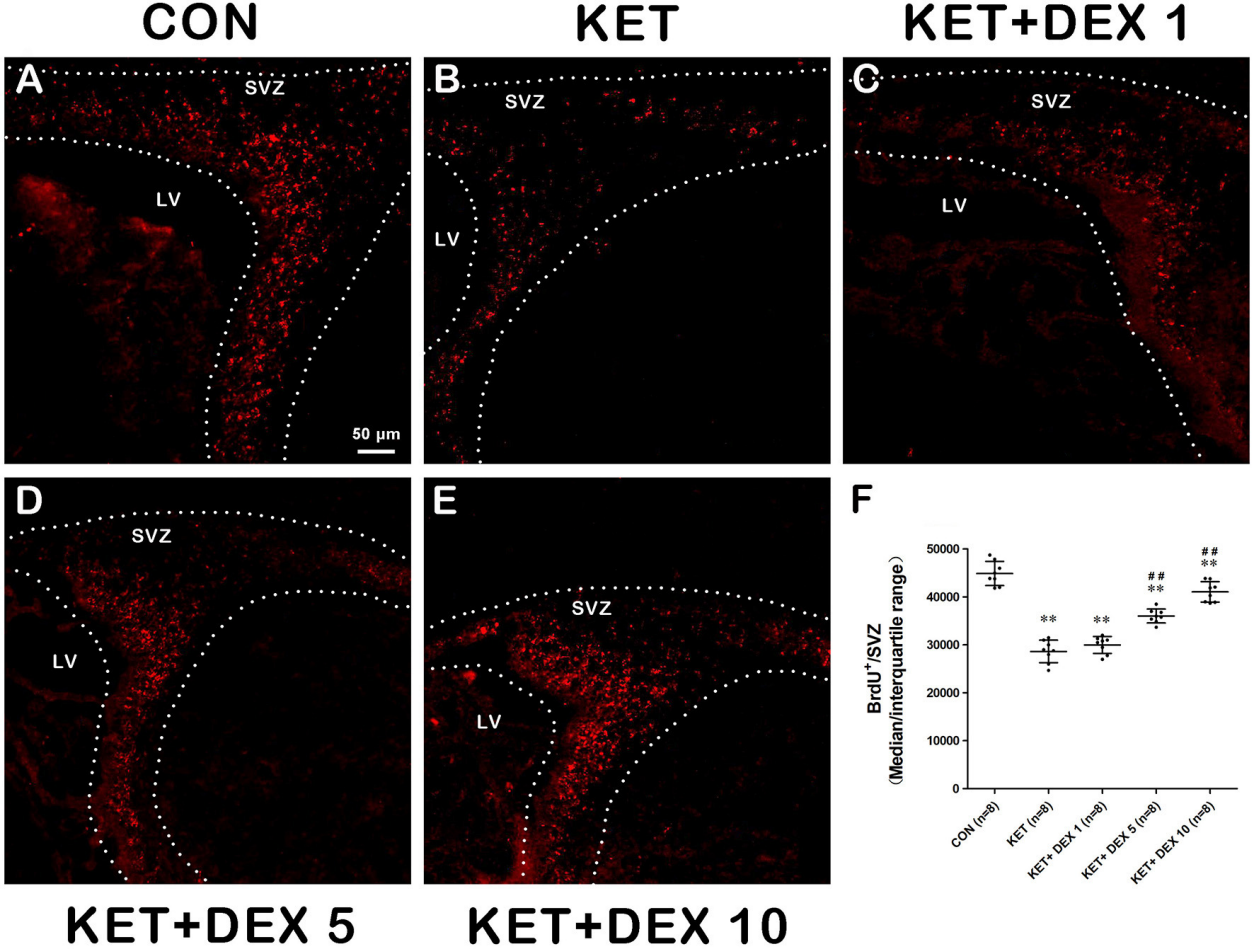
The effects of ketamine (KET) and dexmedetomidine (DEX) on the proliferation of neural stem cells (NSCs) in the SVZ. Neonatal rats were pretreated with different doses of DEX *via* intraperitoneal injection prior to ketamine anesthesia for 30 min. **(A–E)** Representative images of NSC proliferation (BrdU staining, red) were captured using a laser scanning confocal microscope (magnification ×200, scale bar: 50 μm). **(F)** The number of BrdU^+^ cells per SVZ in the different groups is shown. Data are presented as the means ± SD (eight tissue sections per group). **p < 0.01 compared with the Control group; ^##^*p* < 0.01 compared with the KET group. SVZ, subventricular zone; LV, lateral ventricle.

BrdU immunofluorescence staining was performed to evaluate the proliferation of NSCs in the SVZ. When 100 mg/kg BrdU was injected immediately after anesthesia in PND-7 rats, we observed a substantial reduction in the number of BrdU^+^ cells in the SVZ of the ketamine group (median [IQR]: 28900 [26500-30750]) compared to the control group (median [IQR]: 44435 [42450-47408], shown in Figures 1A,B, *n* = 8, *p* < 0.01). Thus, neonatal ketamine exposure inhibited NSC proliferation in the SVZ.

**Figure 2:**
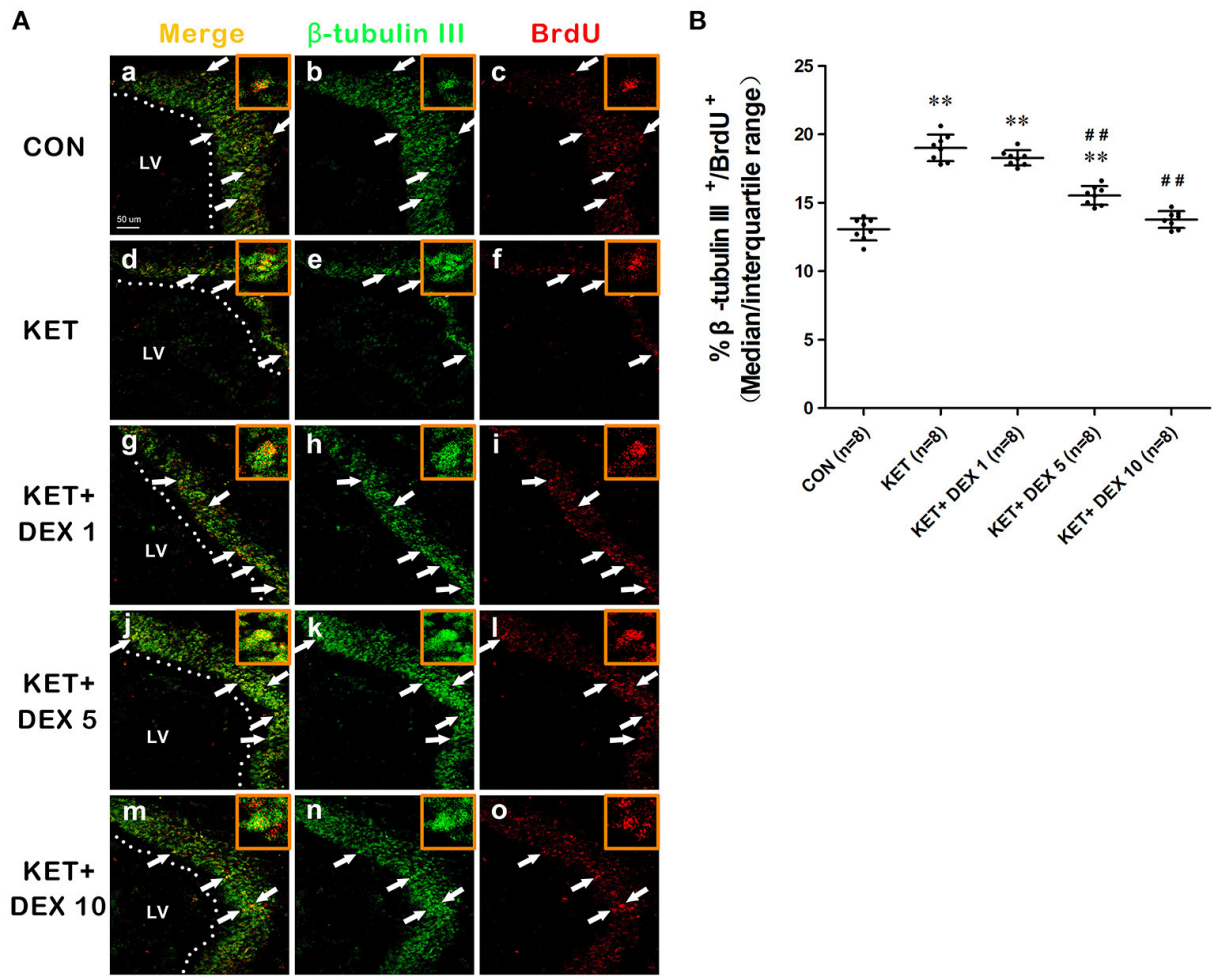
The effects of ketamine (KET) and dexmedetomidine (DEX) on the neuronal differentiation of neural stem cells (NSCs) in the SVZ. Neonatal rats were pretreated with different doses of DEX *via* intraperitoneal injection prior to ketamine anesthesia for 30 min. **(A)** Representative images of neuronal differentiation (BrdU, red; β-tubulin III, green) were captured using a laser scanning confocal microscope (magnification ×400, scale bar: 50 μm). The arrows point to postively labeled cells. **(B)** The proportion of β-tubulin III^+^/BrdU^+^ cells per SVZ in the different groups is shown. Data are presented as the means ± SD (eight tissue sections per group). **p < 0.01 compared with the Control group; ^##^*p* < 0.01 compared with the KET group. SVZ, subventricular zone; LV, lateral ventricle.

NSCs have the ability to differentiate into neurons, and the early neuronal marker associated with differentiation is β-tubulin III. BrdU^+^ cells coexpressing the neuronal marker β-tubulin III were analyzed to assess the neuronal differentiation of NSCs. In the present study, the proportion of β-tubulin III^+^/BrdU^+^ cells among BrdU^+^ cells was increased in the ketamine group (median [IQR]: 19.05% [18–19.725%]) compared to control animals (median [IQR]: 13.15% [12.475–13.7%]) at 24 h after the BrdU injection (Figures 2A,B, *n* = 8, *p* < 0.01).

**Figure 3:**
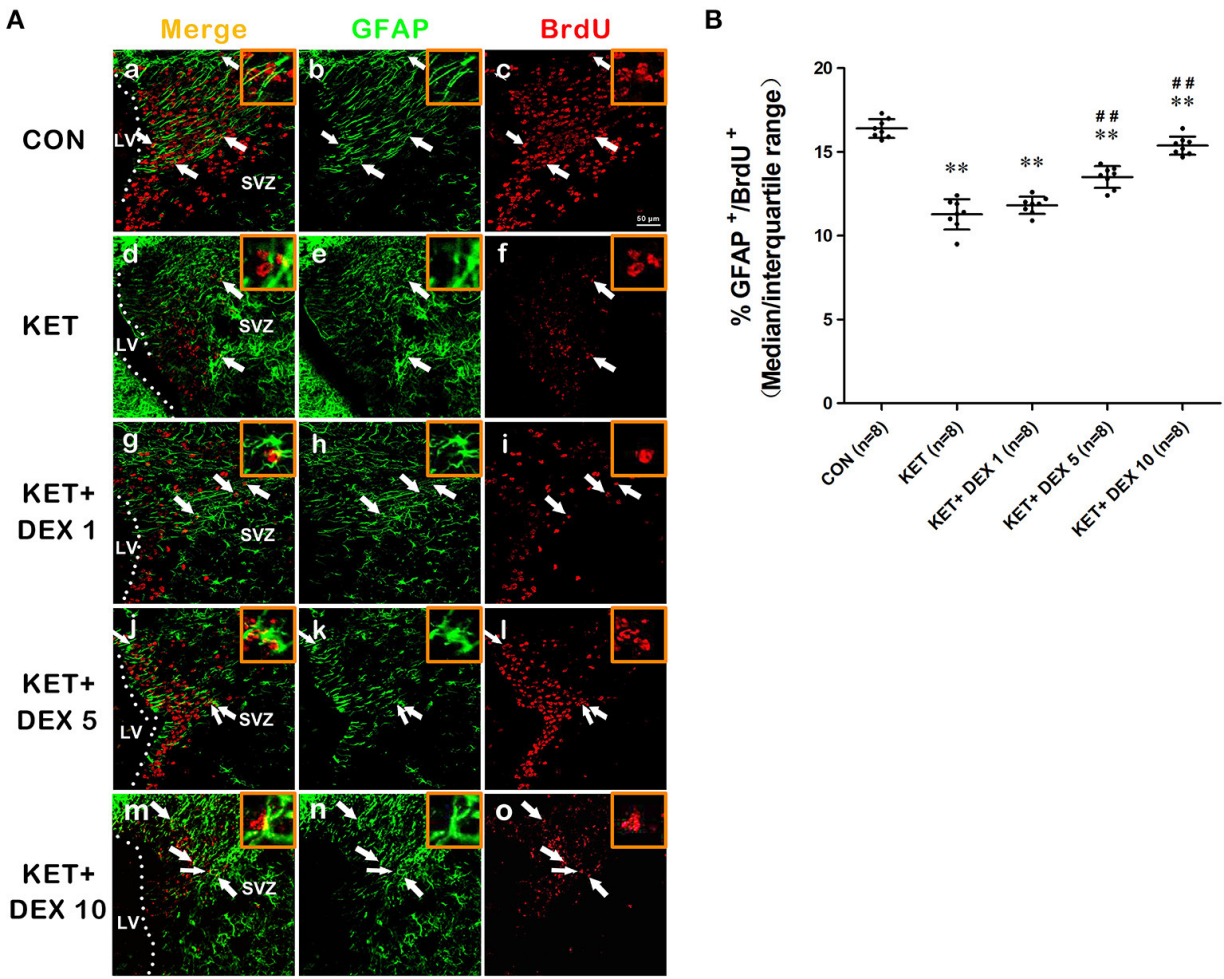
The effects of ketamine (KET) and dexmedetomidine (DEX) on the astrocytic differentiation of neural stem cells (NSCs) in the SVZ. Neonatal rats were pretreated with different doses of DEX *via* intraperitoneal injection prior to ketamine anesthesia for 30 min. **(A)** Representative images of astrocytic differentiation (BrdU, red; GFAP, green) were captured using a laser scanning confocal microscope (magnification ×400, scale bar: 50 μm). A portion of BrdU postive cells expressed GFAP in the cytoplasm. The GFAP^+^/BrdU^+^ cells implies that BrdU and GFAP form close appositions, but they are not co-located. The arrows point to postively labeled cells. **(B)** The proportion of GFAP^+^/BrdU^+^ cells per SVZ in the different groups is shown. Data are presented as the means ± SD (eight tissue sections per group). **p < 0.01 compared with the Control group; ^##^*p* < 0.01 compared with the KET group. SVZ, subventricular zone; LV, lateral ventricle.

NSCs also have the potential to differentiate into astrocytes. The proportion of BrdU^+^ cells coexpressing GFAP was calculated to assess the astrocytic differentiation of NSCs. In the present study, we observed that a portion of BrdU postive cells expressed GFAP in the cytoplasm. The GFAP^+^/BrdU^+^ cells in the Figure 3A implies that BrdU and GFAP form close appositions, they are not co-located. Our results revealed that the proportion of GFAP^+^/BrdU^+^ cells among BrdU^+^ cells was significantly greater in control rats (median [IQR]: 16.3% [15.925–16.925%]) than in ketamine-anesthetized rats (median [IQR]: 11.35% [10.775–11.975%], Figure 3B, *n* = 8, *p* < 0.01) at 24 h after the BrdU injection. Based on these findings, neonatal ketamine exposure significantly promoted neuronal differentiation and attenuated the astrocytic differentiation of NSCs in the SVZ.

### Dexmedetomidine Protects Against Ketamine-Induced Damage to the Proliferation and Differentiation of NSCs

Next, we investigated the effect of dexmedetomidine (DEX) on the ketamine-induced disruption of the proliferation and differentiation of NSCs in the SVZ using immunofluorescence staining. As shown in Figures 1D,E, pretreatment with 5 μg/kg DEX or 10 μg/kg DEX before ketamine anesthesia significantly increased the number of BrdU^+^ cells in the SVZ compared to the ketamine group (median [IQR]: 35900 [35025–36950] in the 5 μg/kg DEX plus ketamine group, median [IQR]: 41100 [38925–43368] in the 10 μg/kg DEX plus ketamine group, and median [IQR]: 28900 [26500–30750] in the ketamine group, *n* = 8, *p* < 0.01).

Moreover, compared with the ketamine group, pretreatment with 5 μg/kg DEX or 10 μg/kg DEX before ketamine anesthesia significantly decreased the proportion of β-tubulin III^+^/BrdU^+^ cells in the SVZ compared with the ketamine treatment (median [IQR]: 15.6% [14.85%−16.05] in the 5 μg/kg DEX plus ketamine group, median [IQR]: 13.85 [13.125–14.175] in the 10 μg/kg DEX plus ketamine group, and median [IQR]: 19.05% [18%−19.725%] in the ketamine group, *n* = 8, *p* < 0.01, Figures 2A,B). In addition, DEX pretreatment dose-dependently ameliorated the reduction in the proportion of GFAP^+^/BrdU^+^ cells in the SVZ caused by ketamine anesthesia (median [IQR]: 13.7% [12.88%−13.98] in the 5 μg/kg DEX plus ketamine group, median [IQR]: 15.35 [14.93–15.68] in the 10 μg/kg DEX plus ketamine group, and median [IQR]: 11.35% [10.775%−11.975%] in the ketamine group, *n* = 8, *p* < 0.01, Figures 3A,B). However, the 1 μg/kg DEX pretreatment did not exert a protective effect on the ketamine-induced disruption of NSC proliferation and differentiation (*p* > 0.05, Figures 1F, 2B, 3B).

In summary, moderate- and high-dose DEX pretreatments alleviated ketamine-induced disturbances in the proliferation and differentiation of NSCs in the SVZ.

### Dexmedetomidine Alleviates Olfactory Cognitive Dysfunction in the Adult Stage Caused by Neonatal Ketamine Exposure

**Figure 4:**
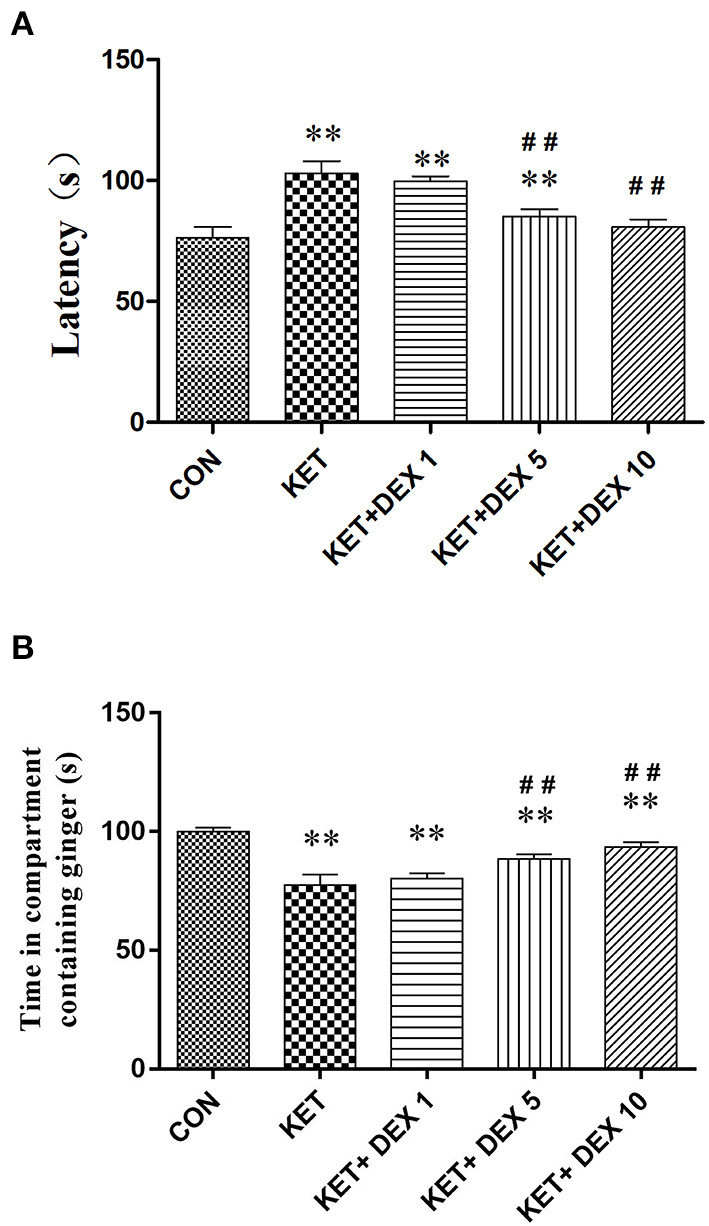
The effects of ketamine and dexmedetomidine (DEX) exposure during development on performance on olfactory behavioral tests in the adult stage. The buried food test **(A)**. The latency to find the feed within 5 min in the ketamine group was significantly longer than that in the control group. The longer latency to find hidden feed induced by ketamine was partially reversed by dexmedetomidine (DEX) in a dose-dependent manner. The olfactory memory test **(B)**. The time that rats stayed in the compartment containing ginger essential oil was significantly decreased in the ketamine group compared with the control group. The olfactory memory dysfunction induced by ketamine was partially reversed by dexmedetomidine (DEX) in a dose-dependent manner. The data are presented as the means ± SD (*n* = 10 per group). **p < 0.01 compared with the Control group; ^##^*p* < 0.01 compared with the KET group.

In the buried food test, the latency to find hidden feed in the ketamine group (102.95 ± 5.12 s) was significantly longer than that in the control group (76.42 ± 4.37 s) (Figure 4A). In the olfactory memory test, the time that rats stayed in the compartment containing ginger essential oil was significantly decreased in the ketamine group (77.59 ± 4.29 s) compared with the control group (100.07 ± 1.59 s) (Figure 4B). Our findings indicated that neonatal ketamine exposure may induce olfactory cognitive dysfunction in the adult stage.

We next investigated the protective effect of different doses of DEX on the impairment of olfactory function induced by neonatal ketamine exposure. In the buried food test, we found that the longer latency to find hidden feed induced by ketamine was partially reversed by DEX 5 (90.46 ± 2.93 s in the 5 μg/kg DEX plus ketamine group, vs. ketamine group, *p* < *0.01*) and was completely reversed by DEX 10 (80.79 ± 3.11 s in the 10 μg/kg DEX plus ketamine group, vs. control group, *p* > *0.05*) (Figure 4A). In the olfactory memory test, the time that rats stayed in the compartment containing ginger essential oil was notably increased in the KET+DEX 5 (88.51 ± 1.89 s, *p* < *0.01*) and KET+DEX 10 groups (96.96 ± 2.7 s, *p* < *0.01*) compared with the KET group, and no significant difference was observed between the CON and KET+DEX 10 groups. Meanwhile, 1 μg/kg DEX had no protective effect on olfactory cognitive dysfunction induced by ketamine (*p* > *0.05*) (Figure 4B).

## Discussion

Ketamine is a dissociative anesthetic that is commonly used in pediatric anesthesia. Based on accumulating preclinical evidence, ketamine may cause neurodegeneration and neuroapoptosis in the developing brain and precipitate significant long-term cognitive sequelae in rodents and non-human primates. Epidemiological evidence has indicated that long-term neurotoxicity may ensue following prolonged and/or repeated exposure to ketamine in early life. The U.S. Food and Drug Administration (FDA) issued a warning about prolonged and/or repeated exposure to general anesthetics and their potential negative effects on the developing brains of children (20).

The purpose of this study was to investigate the effect of prolonged and/or repeated exposure to general anesthetics on the developing brain and explore potential protective approaches. We performed dose-dependent studies and selected four doses of ketamine, including 10, 20, 40, and 60 mg/kg. Four injections of 10 or 20 mg/kg ketamine at 1 h intervals only obviously reduced the movement of animals, while the righting reflex still existed in some animals. In addition, only a small percentage of neonatal rats survived after the end of four injections of 60 mg/kg ketamine at 1 h intervals. Finally, four injections of 40 mg/kg ketamine at 1 h intervals exerted a satisfactory anesthesia effect, and all animals survived after anesthesia. Therefore, four injections of 40 mg/kg ketamine at 1 h intervals were used to construct a model of multiple anesthesia events during the neonatal period in the present experiment.

Neurogenesis is an important process that occurs in multiple brain regions, particularly in the hippocampal dentate gyrus (DG) and subventricular zone (SVZ), from the embryonic to adult stages (17, 18). The vast majority of NSCs are in a mitotically active state in the developing brain, and the balance between the mitosis and quiescence of NSCs is crucial to maintain the balance between the types and numbers of cells in the brain, which are the origins of neurons and glial cells. In rodents, a large number of neurons are established during gestation and throughout the first 21 postnatal days, which play critical roles in the formation of neural networks. Postnatal neurogenesis, particularly in the hippocampal DG and SVZ, was recently appreciated to be critically important for brain function. As shown in our previous study, neonatal ketamine exposure disrupts the proliferation and differentiation of NSCs in the developing hippocampal DG and causes hippocampus-dependent spatial memory dysfunction in the adult stage (6).

During the developmental period of SVZ neurogenesis, newly generated neurons migrate along the rostral migratory stream (RMS) to the olfactory bulb (OB), where the neurons are incorporated into existing neural circuits and play a crucial role in long-term olfactory recognition memory (21, 22). The disruption of SVZ neurogenesis has the potential to alter the formation of neural circuits (23) and has been linked behaviorally and biochemically to psychiatric disorders, such as major depression, schizophrenia and olfactory cognitive dysfunction, in adulthood (24, 25). Based on the findings from the present study, repeated ketamine exposure (40 mg/kg × 4 injections) during the developmental stage disrupts neurogenesis, including the significant inhibition of NSC proliferation and astrocytic differentiation and the induction of neuronal differentiation in the SVZ. These results were consistent with our previous findings showing that ketamine affects the fates of NSCs in the hippocampal DG and SVZ (6, 26).

Olfactory function mainly includes olfactory detection and olfactory memory (27). A buried food experiment was used to test the olfactory detection ability of rats (28). Some researchers modified the experimental method by using the normal diet of mice as buried food to further optimize the experimental results (29). The olfactory memory experiment tested olfactory cognitive function by mixing food with smells. In the non-fasting condition, no significant difference in the preference between cinnamon and ginger was observed. Under fasting conditions, food and ginger were placed in the same compartment, the rats were allowed to explore the food in the experimental device, and a connection between food and ginger was established in the brain. In the testing phase, the rats preferred to stay in the ginger compartment after the food was removed, based on their olfactory memory (30). In the present study, the buried food test and olfactory memory test were sensitive to the effects of neonatal ketamine exposure on olfactory function in the adult stage. Notably, ketamine clearly impaired the ability of rats to detect “familiar” odors and olfactory cognitive memory. In the ketamine-induced neurotoxicity model in 7-day-old rats, our findings suggested that abnormalities in the proliferation and differentiation of NSCs in the SVZ may be closely associated with olfactory cognitive dysfunction in the adult stage.

Neuroprotective measures are constantly being explored for general anesthesia administered to pediatric patients, and anesthetics with neuroprotective effects have been widely investigated to avoid the adverse neurological complications of conventional anesthetics. In contrast to ketamine, the α_2_-adrenoceptor agonist dexmedetomidine (DEX) is an adjuvant anesthetic that has been extensively studied in recent years, as it produces sedative, analgesic, sympatholytic and anxiolytic effects during the perioperative period. To date, at least three different α_2_ receptors (α_2A_, α_2B_ and α_2C_) have been identified based on pharmacological and molecular biological probes (31). The α_2A_ subtype correlates with sedative, analgesic, neuroprotective and sympatholytic effects (32). Previous studies have shown that the molecular mechanism of DEX-mediated anesthesia is to suppress noradrenergic neuronal firing by acting on the α_2A_ adrenergic receptors of the locus ceruleus in the brainstem (33), which leads to a loss of wakefulness *via* activation of an endogenous sleep-promoting pathway (34). The role of DEX in neonatal intensive care medicine and pediatric anesthesia has been an interesting research topic in recent years. In preclinical studies, DEX has attracted the attention of researchers and clinicians because it exerts neuroprotective effects on hippocampal neurogenesis and neuronal plasticity in a model of neonatal brain injury (35).

In the field of anesthetic-induced developmental neurotoxicity, DEX has been proven to exert neuroprotective effects on anesthetic-induced neurodegeneration and neuroapoptosis in the developing brain (13, 14, 36–38). According to a recent *in vitro* study, DEX protects NSCs in the embryonic cortex from ketamine-induced injury (39). Although DEX has been extensively studied as a clinical adjuvant anesthetic, little is known about its neuroprotective effects on the proliferation and differentiation of NSCs after neonatal ketamine exposure in animal models. In the clinic, pediatric patients particularly have higher requirements and a better tolerance for DEX (40, 41). In the present study, the dose of DEX was based on clinical concentrations that had been used in children (1 μg/kg) (42) or had been shown to exert neuroprotective effects on other animal models (5 μg/kg and 10 μg/kg) (43, 44). In the present study, one key finding was the substantial reversal of the ketamine-induced disruption of the proliferation and differentiation of NSCs in the SVZ by a single pretreatment with DEX at intermediate (5 μg/kg) or high doses (10 μg/kg). However, the administration of low-dose DEX (1 μg/kg) did not produce potential neuroprotective effects. In the olfactory cognitive test, treatment with the intermediate or high concentration of DEX reduced the prolonged latency to find the hidden feed induced by ketamine, and significant differences in the performance of remembering the familiar odor were not observed compared with control animals. However, the low-dose administration of DEX (1 μg/kg) did not diminish olfactory cognitive dysfunction induced by ketamine. These findings may provide a basis for the use of a combination of DEX and ketamine in pediatric anesthesia.

This study has several limitations. Although the present findings indicated that DEX pretreatment alleviated the ketamine-induced disruption of NSC proliferation and differentiation in the SVZ, the potential mechanisms have not been elucidated. As a second messenger, Ca^2+^ can activate a series of Ca^2+^-dependent protein kinase C (PKC), which plays an important role in modulating a variety of biological responses including the regulation of cell growth. In our previous *in vitro* study, suppressing Ca^2+^-PKCα-ERK1/2 signaling pathway may be involved in the inhibitory effect of ketamine on hippocampal NSC proliferation (45). Previous studies had revealed that the α_2_-adrenoceptor subtype expressed in the NSC and it has been confirmed to mediate the development of fetal cortex (39, 46). Therefore, our future studies will include exploring whether DEX pretreatment exerted a protective effect on the disruption of NSC proliferation and differentiation in the SVZ caused by neonatal ketamine exposure through regulating the calcium signaling pathway *in vivo* study. In addition, the present results only revealed the alterations in NSC proliferation and differentiation in the SVZ at 24 h after ketamine exposure and the relevant information about NSC proliferation and differentiation in the SVZ at 7 days and 14 days postintervention were not provided in this study, a better approach will be to obtain additional results and relevant information in a future study.

## Conclusions

The neuroprotective effect of DEX has been an interesting topic of neonatological and pediatric anesthetic research in recent years. In conclusion, the animal experiment preliminarily confirmed that the moderately high- (5 μg/kg) or high-dose (10 μg/kg) DEX pretreatment in neonatal period alleviated the disruption of NSC proliferation and differentiation in the SVZ induced by ketamine during development. Moreover, the moderately high- or high-dose DEX pretreatment in neonatal period exerted a protective effect on long-term olfactory memory dysfunction caused by neonatal ketamine exposure. Before its safe and efficient application in clinical pediatric anesthesia, the potential neuroprotective mechanisms underlying the effect of DEX on ketamine-induced neurotoxicity require further experimental and clinical investigations.

## Data Availability Statement

The raw data supporting the conclusions of this article will be made available by the authors, without undue reservation.

## Ethics Statement

The animal study was reviewed and approved by Institutional Animal Care and Use Committee of the Nanjing Medical University. Written informed consent was obtained from the owners for the participation of their animals in this study.

## Author Contributions

HH, HS, and PP: conceived and designed the experiments. HS, PP, GW, and JW: performed the experiments. HS, PP, HH, and YW: data analysis and interpretation. YW: contributed reagents/materials/analysis tools. HS, PP, and HH: manuscript preparation. All authors read and approved the final manuscript.

## Conflict of Interest

The authors declare that the research was conducted in the absence of any commercial or financial relationships that could be construed as a potential conflict of interest.

## Publisher's Note

All claims expressed in this article are solely those of the authors and do not necessarily represent those of their affiliated organizations, or those of the publisher, the editors and the reviewers. Any product that may be evaluated in this article, or claim that may be made by its manufacturer, is not guaranteed or endorsed by the publisher.

## Abbreviations

NSCs: neural stem cells
KET: ketamine
DEX: dexmedetomidine
SVZ: subventricular zone
PND-7: Postnatal day 7
BrdU: 5-bromo-2-deoxyuridine
DG: dentate gyrus
RMS: rostral migratory stream
OB: olfactory bulb.
